# Self‐truncated sampling produces more moderate covariation judgment and related decision than descriptive frequency information: The role of regressive frequency estimation

**DOI:** 10.1002/pchj.703

**Published:** 2023-12-17

**Authors:** Xuhui Zhang, Junyi Dai

**Affiliations:** ^1^ Department of Psychology and Behavioral Sciences Zhejiang University Hangzhou China

**Keywords:** contingency table, covariation judgment, decision, regressive frequency estimation, self‐truncated sampling

## Abstract

Covariation judgment underlies a diversity of psychological theories and influences various everyday decisions. Information about covariation can be learned from either a summary description of frequencies (i.e., contingency tables) or trial‐by‐trial experience (i.e., sampling individual instances). Two studies were conducted to investigate the impact of information learning mode (i.e., description vs. self‐truncated sampling) on covariation judgment and related decision. In each trial under the description condition, participants were presented with a contingency table with explicit cell frequencies, whereas in each trial under the self‐truncated sampling condition, participants were allowed to determine when to stop sampling instances and thus the actual sample size. To eliminate sampling error, an other‐yoked (i.e., between‐subject) design was used in this research so that cell frequencies shown in a trial under the description condition were matched with those experienced in a trial under the self‐truncated sampling condition. Experiment 1 showed that the self‐truncated sampling condition led to more moderate covariation judgments than the description condition (i.e., a description–experience gap). Experiment 2 demonstrated further that the same gap extended to covariation‐related decisions in terms of relative contingent preference (RCP). Regressive frequency estimation under self‐truncated sampling appeared to underlie the consistent gaps found in the two studies, whereas the impact of regressive diagnosticity (i.e., the same sample of instances was viewed as less diagnostic under description than under self‐truncated sampling) or simultaneous overestimation and underweighting of rare instances under experience was not supported by the observed data. Future research might examine alternative accounts of the observed gaps, such as the impacts of belief updating and stopping rule under self‐truncated sampling.

## INTRODUCTION

Understanding how people interpret the concept of covariation and make relevant judgments and decisions is of both theoretical and practical significance. The concept of covariation plays a pivotal role in a wide range of psychological theories (for reviews, see Alloy & Tabachnik, 1984; Mata, 2016; Shaklee, 1983). In addition, covariation judgment is indispensable for scientific reasoning aimed at discovering causal relationships among different variables, for clinical diagnoses heavily relying on learned relationships between symptoms and disorders, and for everyday decisions based on knowledge about associations between different events. As Crocker (1981) famously claimed, such knowledge “enables individuals to explain the past, control the present, and predict the future” (p. 272).

**FIGURE 1:**
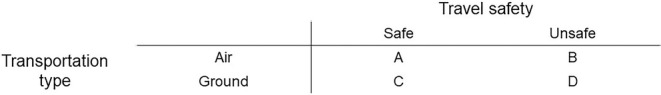
A 2×2 contingency table wherein A, B, C, and D represent the respective frequencies of the four possible combinations of the values of the two dichotomous variables.

A variety of real decisions can be understood from the perspective of covariation judgment. For example, many people are so concerned with the safety of air travel that they have an unjustified preference for ground transport over air transport. To make a wise and well‐informed choice between ground and air transport, one might assess the covariation between transportation type and travel safety. This assessment might be performed by calculating the correlation between the two dichotomous variables using information from a contingency table with respective frequencies (i.e., A, B, C, and D in Figure 1) of the four possible combinations of variable values. In addition, $\Phi =\frac{\mathrm{AD}-\mathrm{BC}}{\sqrt{\left(A+B\right)\left(C+D\right)\left(A+C\right)\left(B+D\right)}}$ is often used as a measurement index of the direction and degree of correlation between the two variables. A zero value of the index indicates no correlation between the two variables, that is, transportation type is unrelated to travel safety. Consequently, type of transportation does not need to be considered while making a safety‐related decision. When the Φ index assumes a non‐zero value, however, different types of transportation are associated with different levels of travel safety. In this case, air transport is safer and thus preferable to ground transport if Φ>0, whereas ground transport is safer and thus preferable to air transport if Φ<0.

Several studies, however, have revealed that people's covariation judgment might deviate from what is suggested by the Φ index or other normative indices of correlation. An illustrative example comes from the empirical finding of illusory correlation, that is, people tend to mistakenly perceive an association between two unrelated events (e.g., Chapman & Chapman, 1969; Hamilton et al., 1985). Similarly, a few studies also demonstrated that laypeople without statistical training (e.g., student nurses) did not consider all relevant information when making covariation judgment. This led investigators to conclude that people did not fully comprehend the concept of contingency (Jenkins & Ward, 1965) and correlation (Smedslund, 1963), or they assumed a pessimistic attitude toward human ability of covariation perception (e.g., Nisbett & Ross, 1980).^1^

### Factors Influencing Covariation Judgment

Previous research has demonstrated several factors that might render people's judgment different from that suggested by normative measures, including the unequal weighting of the four cell frequencies in a contingency table, pseudocontingency, memory demands, and prior expectation. Specifically, co‐occurrence of two events in a correlational context or that of the cause and effect in a causal context tended to attract more attention than other possible combinations, leading to overweighting of the corresponding cell information and potentially illusory correlations (e.g., Kao & Wasserman, 1993; Wasserman et al., 1990). Furthermore, people were inclined to rely on univariate base rates as assessed in one or more ecological context rather than the joint observations of bivariate pairs to make a covariation assessment (e.g., Fiedler et al., 2009; Fiedler & Freytag, 2004; Vogel et al., 2013). Covariation judgment might also deviate from a normative measure (i.e., Δp) under high memory demand due to poor recall of the cell frequencies and a tendency to use simpler judgment strategies (e.g., Shaklee & Mims, 1982). Finally, prior expectation could influence covariation judgment as well (e.g., Alloy & Tabachnik, 1984; Hamilton & Gifford, 1976), potentially through its impacts on several underlying steps of the judgment process (Crocker, 1981).

An equally important but less studied factor that might influence covariation judgment is the information learning mode (e.g., Beyth‐Marom, 1982; Ward & Jenkins, 1965). Contingency information might be learned either in summarized format as cell frequencies shown in a contingency table (i.e., a description condition; e.g., Shaklee & Tucker, 1980) or from trial‐by‐trial observations of variable values of individual instances (i.e., an experience condition; e.g., Arkes & Harkness, 1983; Smedslund, 1963). In real life, people might make covariation judgment and related decision under description to leverage the unambiguous cell information it provides. However, such information tends to be difficult to obtain and thus people oftentimes have to rely on experience for such judgment and decision (Fleig et al., 2017). Therefore, it is valuable to examine whether different information learning modes would lead to distinct covariation judgment and related decision. If so, information learning mode might be manipulated as a means to adjust covariation judgment and related decision for various purposes. For example, if one learning mode tends to produce higher covariation judgments between hard work and success, then we can use this mode to promote hard work.

A majority of previous studies on covariation judgment have adopted the experience condition for information learning. In such studies, the amount of experienced information (i.e., the sample size) was determined either by the experimenter in advance (i.e., externally determined sampling) or by the participant who could decide when to stop sampling and make a judgment (i.e., self‐truncated sampling; Fiedler & Kareev, 2006).^2^ Most research on experience‐based covariation judgment has adopted the externally determined mode (e.g., Mercier & Parr, 1996; Ward & Jenkins, 1965; Yates & Curley, 1986), with a few exceptions with the self‐truncated mode (Fiedler & Kareev, 2006; Fiedler et al., 2010).

As research in other domains of judgment and decision‐making, several studies on covariation judgment have revealed differences between the description and experience conditions (i.e., a description–experience gap). For example, Kao and Wasserman (1993) found that, for noncontingent variables, learning covariation information by externally determined sampling resulted in non‐zero contingency judgment and more unbalanced use of cell information than learning from description. Similarly, Hamilton et al. (1985) showed that externally determined sampling led to illusory correlation but not the description condition.

### Potential Gaps between the Description and Self‐Truncated Sampling Conditions

Although previous research has produced many insights about how people inferred about covariation and why the resultant judgments might deviate from normatively appropriate measures, two critical issues remain to be considered for a better understanding of everyday covariation judgment and related decision. First, it is arguable that experience‐based covariation judgments in real life are more likely to be made under a self‐truncated mode. For example, due to limited cognitive capacity or a need for quick response, one might stop information search early rather than raking through all possible information sources for an optimal judgment. Second, a covariation judgment is usually made for other judgments and decisions rather than for its own sake (e.g., Crocker, 1981). Therefore, the examination of covariation judgment should not be the end of the endeavor. Combined, these two issues suggest that it is important to treat the self‐truncated sampling as a major mode of experience‐based learning and examine the corresponding description–experience gaps in both covariation judgment and related decision. To our best knowledge, however, only a few studies have examined covariation judgment under self‐truncated sampling, not to mention a direct comparison between self‐truncated sampling and the description condition regarding covariation judgment and related decision.

Several lines of existing research suggested such description–experience gaps, but the predicted directions of difference did not always coincide with one another. On the one hand, research on the impact of memory demand on covariation judgment (e.g., Arkes & Harkness, 1983; Shaklee & Mims, 1982) and regressive frequency estimation under externally determined sampling condition (e.g., Fiedler & Unkelbach, 2014) implied that self‐truncated sampling would lead to more moderate covariation judgments and weaker preferences in related decisions. Besides relying on memories of observed instances as in externally determined sampling for covariation judgment and related decision, self‐truncated sampling also entails consecutive decisions about whether to stop an information search. Consequently, it tends to impose an even higher cognitive load than the externally determined sampling and thus is more likely to produce regressive frequency estimations relative to the experienced frequencies. If participants rely on such estimated frequencies under the self‐truncated sampling condition and experienced frequencies that are summarized and explicitly presented under the description condition for covariation judgments and related decisions (Crocker, 1981), the regressive estimation under self‐truncated sampling would naturally lead to more moderate results.

On the other hand, self‐truncated sampling might instead produce more extreme covariation judgments and decisions than the description condition according to other existing research. Recent research on impression formation (Prager & Fiedler, 2021; Prager et al., 2018) suggested that self‐truncated sampling tended to produce more extreme judgment than externally determined sampling, no matter whether the self‐truncated sample was later presented to the same person (i.e., the self‐yoked condition) or another person (i.e., the other‐yoked condition) under the externally determined mode. Specifically, self‐truncated sampling tended to produce small samples with clear‐cut, converging, and extreme information that facilitated a sufficiently confident judgment. However, when presented in an externally determined mode later, the same sample would appear less diagnostic due to fluctuations in subjectively perceived diagnosticity, leading to more regressive and thus weaker judgment. Note that covariation judgment is highly related to impression formation (e.g., Hamilton et al., 1985; Hamilton & Rose, 1980), and that the sample size and content under the description condition for covariation judgment are also externally determined. Therefore, the diagnosticity effect revealed in impression formation research might also render covariation judgment under self‐truncated sampling more extreme than that under description. As a result, whether self‐truncated sampling would lead to more moderate or extreme covariation judgment appears to depend on the impact of regressive frequency estimation under self‐truncated sampling relative to that of regressive diagnosticity under description.

Research on the description–experience gap in risky choice provided yet another theoretical foundation for analyzing description–experience gaps in covariation judgments and decisions. A typical pattern revealed in empirical studies was that rare events were overweighted under description but underweighted or at least less overweighted under experience (Wulff et al., 2018). Such differential weighting of rare events was commonly invoked to account for the observed gap in risky choice. Similar mechanisms might also play a role in covariation judgment. For example, for a pair of positively correlated dichotomous variables, instances in cell B and C tend to be rare ones, especially when each variable has an approximately balanced distribution. If rare instances in a covariation judgment task are also overweighted under description but underweighted under experience, judgments under description shall be more moderate than those under experience. In other words, there should be a description–experience gap in covariation judgment with a direction opposite to that suggested by regressive frequency estimation. The same also applies to negatively correlated variables.

Even if regressive frequency estimation under self‐truncated sampling exerts a higher impact than other opposing factors in shaping description–experience gap in covariation judgment, the same pattern might not extend to related decisions. The dominating status of regressive frequency estimation means that rare instances under self‐truncated sampling would be overestimated relative to their experienced frequencies. However, a number of studies have shown that, although rare events in an uncertain environment tended to be overestimated under experience (e.g., Hertwig et al., 2004; Wulff et al., 2018), they were usually underweighted in the relevant decisions with regard to their experienced probabilities (e.g., Camilleri & Newell, 2011; Yechiam & Busemeyer, 2006). If rare instances in covariation‐related decisions were indeed underweighted, more extreme decisions might occur under self‐truncated sampling than under description. In summary, although regressive frequency estimation tends to produce more moderate covariation judgments and related decision, previous research also suggested that such a gap might be alleviated or even reversed by opposite impacts of regressive diagnosticity under description and differential weighting of rare instances between the description and experience conditions.

### The Present Research

Given the above analyses, the purpose of the current research was twofold: (1) to examine whether self‐truncated sampling would lead to more moderate or extreme covariation judgments and decisions than the description condition; and (2) to investigate the cognitive underpinnings of such gaps. Note that a yoked design was adopted in this research to control for sampling errors. As a result, any explanation of the observed gaps based on sampling error would be irrelevant.

As mentioned above, previous research has revealed other factors that influenced covariation judgment, including pseudocontingency, unequal weighting of cell information, and prior expectation. Such factors might lead to different results between the self‐truncated sampling and description conditions and thus shall be controlled or explicitly examined. Therefore, approximately balanced distributions of dichotomous variables were used in the current research to control for the impact of pseudocontingency, since it could occur only when base rates of both variables were sufficiently skewed (Fiedler, 2010). Similarly, non‐causal scenarios with symmetric variables were adopted to reduce the impact of unequal weighting of cell information (Beyth‐Marom, 1982). Finally, participants in the reported studies were informed to make covariation judgments and decisions based on the experienced or presented samples (i.e., current situational information; Alloy & Tabachnik, 1984). Nevertheless, their prior expectations were also measured to examine their potential impact.

## EXPERIMENT 1

This study was aimed at investigating whether self‐truncated sampling would lead to more moderate covariation judgments than the description condition due to regressive frequency estimation. As mentioned above, an other‐yoked (i.e., between‐subject) design was adopted in this experiment so that the same primary information was presented under different learning modes to eliminate such error. According to Prager and Fiedler's (2021) work on impression formation, the other‐yoked design was more likely to invoke regressive diagnosticity under the description condition than the self‐yoked design. Consequently, the influence of regressive diagnosticity on covariation judgment, if any, should be facilitated under the current design. Finally, because individual difference in the general tendency to make relatively high versus low covariation judgment might exert a confounding impact on the relevant gap under the between‐subject design, we used a matching procedure to eliminate such an impact.

### Method

#### Participants

Fifty‐four students (16 males; *M*_age_ = 21.0 years, range 18–29 years) from Zhejiang University were recruited for this study. Half of the participants took the self‐truncated condition while the other half took the description condition. Participants were paid with an amount proportional to the average duration of the experiment condition they took. Consequently, participants under the description condition were each paid 15 Chinese Yuan (CNY) and those under the self‐truncated sampling condition were each paid 18 CNY. All participants granted their informed consent before taking the study. This and the following study were approved by the ethics committee of the Department of Psychology and Behavioral Sciences, Zhejiang University.

#### Materials and procedure

This experiment examined a range of moderate objective covariations for a systematic investigation of the description–experience gap in covariation judgment. Specifically, six different covariation levels in Φ value were adopted, with the base rate of each dichotomous variable constrained between 0.4 and 0.6 to reduce the possibility of pseudocontingency (see Table 1 for the Φ values and corresponding cell frequencies). For each participant under the self‐truncated condition, the different levels of objective covariation were randomly paired with a total of 18 non‐causal contexts involving symmetric variables to generate 18 judgment problems under the self‐truncated condition, with each level of objective covariation applied to three contexts. The same pairing was then used under the description condition for a matched participant, with a different random order of the 18 contexts. See Table 2 for an exemplar set of contexts and Supplementary Materials for all the contexts used in this and subsequent study.

**TABLE 1:** Levels of objective covariation in Φ value and the corresponding cell frequencies in the populations of instances used under the self‐truncated sampling condition in Experiment 1.

Φ	A	B	C	D
−0.7	4	20	14	2
−0.5	6	17	13	4
−0.3	8	15	11	6
0.3	11	6	8	15
0.5	13	6	4	17
0.7	19	4	2	15

**TABLE 2:** Pairs of dichotomous variables (with values in parentheses) involved in an exemplar set of contexts presented in each of the reported experiments.

Context	Variable 1	Variable 2
1	Beverage (soy milk/milk)	Food (meat bun/vegetable bun)
2	Level of education (high school/college)	Reading device (paper book/E‐book)
3	Game (reasoning game/shooting game)	Platform (computer/mobile phone)
4	Goods (food/clothing)	Purchasing method (store/E‐commerce)
5	Brand (Nike/Adidas)	Preferred sport (soccer/basketball)
6	Age (children/adult)	Preferred cuisine (Japanese cuisine/Korean cuisine)

The experiment under either information learning mode started with two explanatory examples regarding neutral covariation using a gender‐brand scenario, one of which read as follows:

Some people think that males prefer Audi while females prefer BMW nowadays. Therefore, we would like to know whether there is a relationship between gender and purchased car brand. We have surveyed 500 drivers and here is the result (shown by a 2×2 contingency table as in Figure 1). You can see that 60% of male drivers own an Audi and 60% of female drivers own an Audi. Because these two percentages are the same, we can conclude that there is no relationship between gender and purchased car brand. Can you think of any similar examples?

After reading this and a similar example in which 50 percent of both male and female drivers own an Audi, each participant was required to describe a situation of neutral covariation. If the description was incorrect, the same two examples of neutral covariation would be presented again, and the participant was asked again to describe a situation of neutral covariation. This procedure would continue until a correct description was provided. The experimenter would then proceed to present explanatory examples on positive and negative covariations in a similar way. After presenting the relevant instructions, the experimenter would ask each participant how the level of covariation would change if the percentage of Audi owners among male drivers or BMW owners among female drivers had changed. Again, the answer would be checked to guarantee correct understanding of the relevant concepts. Note that participants were instructed only about what constituted negative, neutral, or positive covariation (i.e., direction of covariation) but not any specific rule to make quantitative judgment concerning the level of covariation. Participants were also reminded that covariation judgment should be bounded between −1 and 1.

##### Self‐truncated sampling condition

Upon providing correct situational descriptions regarding different types of covariation, participants under the self‐truncated sampling condition would move on to accomplish three tasks successively for each context: (1) reporting expected covariation; (2) making covariation judgment under the self‐truncated sampling condition; and (3) estimating frequencies of different instances in the self‐truncated sample. Specifically, after reading a description of the context with a brief introduction of two dichotomous variables, participants should first enter a number between −1 and 1 with two decimal places to indicate their expected covariation regarding the context. Because symmetrical variables were adopted, it was necessary to define what situations signified positive as opposed to negative covariations. Take the scenario concerning the relationship between beverage and food ordered at a canteen as an example. In this case, participants were instructed that a reported value of 1 means that people who order soy milk to drink always order a meat bun to eat and people who order milk to drink always order a vegetable bun to eat. To the contrary, a reported value of −1 means that people who order soy milk to drink always order a vegetable bun to eat and people who order milk to drink always order a meat bun to eat. They were also told that a reported value of 0 means beverage order has no relationship with food order.

**FIGURE 2:**
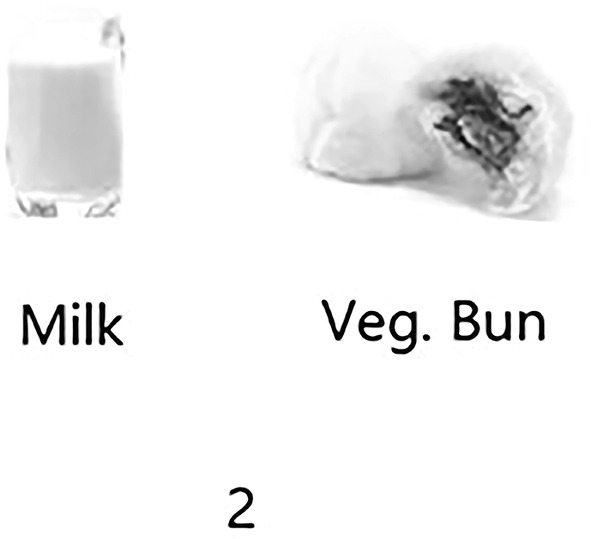
An exemplar visual display under the self‐truncated condition for the canteen order scenario. This display indicated that the second randomly sampled customer ordered milk to drink and a vegetable bun to eat.

After reporting expected covariation for a context, participants would then learn covariation information for the two variables involved in the context by sampling individual instances in a self‐truncated manner and then make covariation judgment. They were instructed that their judgment should be based on the instances they sampled and the response should be a number between −1 and 1 with two decimal places. Each judgment problem under this condition involved a population of 40 instances (see Table 1). Participants could randomly sample the population without replacement and were allowed to stop sampling at their will. Specifically, they were told that they could stop sampling when they felt sufficient information had been collected for a covariation judgment about the relevant context. Note that participants could determine only the number of instances but not which instances to sample at each trial. Participants were also informed in advance that for each problem they could sample at most 40 instances. Since each population of instances was sampled without replacement, the number of observed instances per cell was upper bounded by the cell frequencies in the population. The variable values of each sampled instance were visually displayed for 2000 ms under the experience condition. The inter‐instance‐interval lasted 500 ms, during which a cross was shown at the center of the screen and participants could stop sampling by pressing a key on the keyboard. The cumulative frequency of sampled instances was also shown below the visual display of variable values to reduce memory demand of the judgment task. See Figure 2 for an exemplar visual display.

After reporting their covariation judgment for a context, participants were required to recall the instances they experienced and then enter their frequency estimates into an empty 2×2 contingency table. The four cells were so designated that instances in cells A and D always suggested a positive covariation, whereas those in cells B and C always implied a negative covariation as defined for each context. To prevent participants from paying too much attention to the recall and frequency estimation task, they were warned that covariation judgment was the main task.

##### Description condition

As mentioned earlier, participants under the description condition were first paired with those under the self‐truncated sampling condition to eliminate potential impact of individual difference in covariation judgment on the relevant gap. Specifically, before attending the formal experiment, participants under either condition were required to make four description‐based covariation judgments for scenarios with some moderate objective covariations (i.e., Φ = ±0.6 and ± 0.4). For each participant, the absolute values of their responses would be averaged to produce a measurement of the general tendency to make relatively high versus low covariation judgment. The result was used as a matching index so that, for each matched pair of participants, the difference in the average absolute value was lower than 0.05. The matching operation turned out quite successful in that the two matched groups of participants reported virtually the same distribution of judgments for the four description‐based tasks mentioned above.

After the matching procedure, participants under the description condition first reported expected covariation for each context as those under the self‐truncated sampling condition and then finished the covariation judgment task. Specifically, for each participant who learned covariation information by self‐truncated sampling, their sampled instances were recorded by the experimental program and then summarized as cell frequencies of a 2×2 contingency table. Such a table would be used as the learning material for the matched participant under the description condition. Participants under the description condition were instructed that their judgments should be based on the information shown in the contingency tables and their responses should be numbers between −1 and 1 with two decimal places.

#### Data analysis

A sequence of analyses was conducted to examine whether self‐truncated sampling led to more moderate covariation judgments than the description condition as well as potential cognitive mechanisms underlying this description–experience gap. Due to self‐truncated sampling and the yoked design, the experienced and corresponding described cell frequencies participants learned under respective conditions were likely to differ from cell frequencies in the corresponding populations. Therefore, we first derived Φ values using the learned frequencies and treated the results as major variables for relevant analyses. Such derived variables would be referred to as *experienced/described covariation* hereafter, and they might deviate from the objective covariations shown in Table 1.

With the measures on experienced/described covariation, we ran a systematic comparison of regression models on covariation judgment (hereafter also referred to as judged covariation [JC]) with information learning mode (ILM), experienced/described covariation (EDC), expected covariation (EC), and sample size (SS) as potential predictors. Interactions between information learning mode and the other predictors were also considered to investigate whether such predictors might influence the size of the description–experience gap. Note that information learning mode was directly manipulated in this research, whereas experienced/described covariation was partially determined by objective covariation that was also experimentally manipulated. To the contrary, expected covariation was measured for each participant and context, whereas sample size was determined by participants under the self‐truncated sampling condition. The most complex model considered in this analysis was as follows:

JC∼ILM+EDC+EC+SS+ILM×EDC+ILM×EC+ILM×SS.

If term(s) regarding the main effect of information learning mode or its interaction with other predictor(s) were supported by the analysis, then there must be a description–experience gap in covariation judgment, at least under certain levels of the other predictors.

To further examine the role of regressive frequency estimation in the potential gap, we compared experienced information under self‐truncated sampling with the recalled one in terms of both frequencies per se and Φ values derived from such frequencies. Like experienced and described covariations, Φ values derived from recalled frequencies (i.e., frequency estimates) would be referred to as *recalled covariation* hereafter. To investigate how much of the gap in covariation judgment could be attributed to regressive frequency estimation, we also ran a mediation analysis between information learning mode and judged covariation. The value of the mediator variable was set to equal described covariation under the description condition or recalled covariation under the self‐truncated sampling condition. Finally, we compared described and judged covariations under the description condition as well as the recalled and judged covariations under self‐truncated sampling to shed light on the potential impact of differential weighting of rare events on the gap in covariation judgment. It should be mentioned that all the analyses were conducted at a level of judgment problem and thus each participant contributed multiple data points in each analysis. Consequently, the effective sample sizes were much larger than the number of participants.

All analyses in this and following studies were conducted with a Bayesian approach using the JASP software (JASP Team, 2023) or the R software (R core team, 2022) and its *brms* package (Bürkner, 2017) and *mediation* package (Tingley et al., 2014). For each involved parameter, the default prior distribution of the corresponding software was adopted. The Bayesian approach could offer supporting evidence for either the null or alternative hypothesis and can be easily used to compare models with different levels of complexity (e.g., Rouder et al., 2009). Specifically, the Bayes factor between two models indicates the relative amount of supporting evidence from the data for one model against the other. When the factor differs from 1 substantially (i.e., >3 or <1/3), one can make a statistical inference to accept the better model. Statistical inference can also be made based on a Bayesian 95% credible interval (CI) of the relevant model parameter, which shows a range of credible values that has a 95% probability of covering the true value of the parameter (Kruschke & Liddell, 2018). When such an interval does not include the null value, one can make a statistical inference to accept the corresponding alternative hypothesis (Lindley, 1965).

### Results

#### Description–experience gap

**FIGURE 3:**
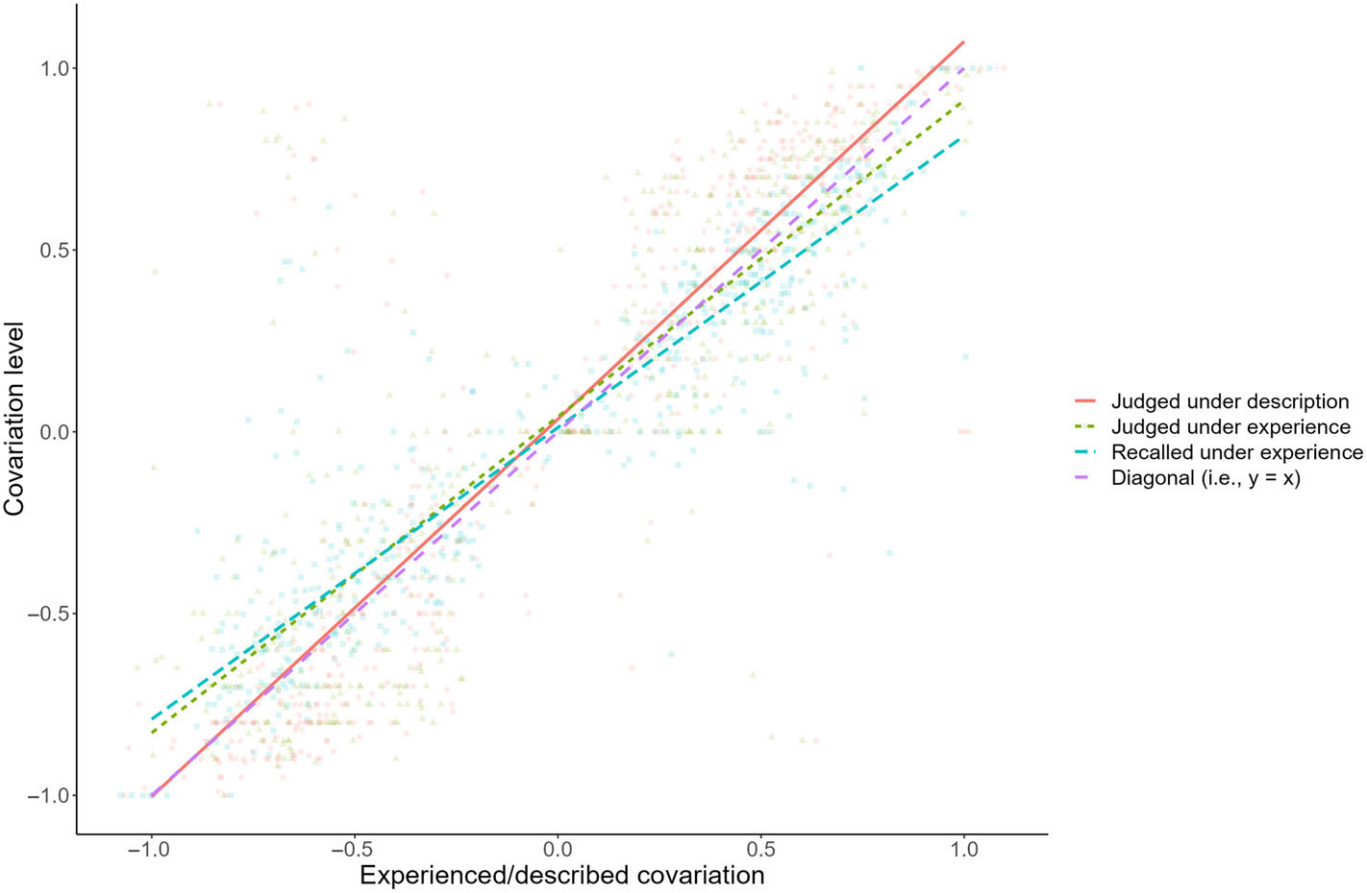
Covariation judgment under description (red elements) and experience (i.e., self‐truncated sampling; green elements) as well as recalled covariation under experience (blue elements) as functions of experienced/described covariation in Experiment 1.

The regression analysis on covariation judgment showed that learning by self‐truncated sampling led to more moderate judgments than learning from described yoked information (i.e., a description–experience gap). Specifically, the best regression model contained only the main terms of information learning mode and experienced/described covariation as well as their interaction (i.e., JC ~ ILM + EDC + ILM × EDC), and it was credibly better than any other regression models considered in this analysis (*BF*s >7.56). Neither expected covariation nor its interaction with information learning mode was supported by the analysis, suggesting that expected covariation did not play a role in covariation judgment in the current study. According to the best model, there was a credible interaction between described/experienced covariation and information learning mode (*BF*_incl_ = 136.18, 95% CI = [−0.242, −0.095]), whereas the main effect of information learning mode was not credible (*BF*_incl_ = 0.036, 95% CI = [−0.034, 0.047]). The interaction suggested that experienced/described covariation had different degrees of influence on covariation judgment between the two learning modes, resulting in the corresponding description‐experience gap. Figure 3 plots the level of judged covariation under both learning modes as well as the level of recalled covariation under self‐truncated sampling against experienced/described covariation in this study. As readily seen, compared with the description condition, self‐truncated sampling resulted in higher covariation judgment given negative experienced/described covariation but lower covariation judgment under positive experienced/described covariation. Furthermore, the more extreme experienced/described covariation was, the larger the description–experience gap grew.^3^ See Table 3 for descriptive statistics of experienced/described, recalled, and judged covariations under different information learning modes and directions of experienced/described covariation.

**TABLE 3:** Descriptive statistics of experienced/described, recalled, and judged covariations under different information learning modes and directions of experienced/described covariation in Experiment 1.

Direction of experienced/described covariation	Type of covariation	Information learning mode	
		Description	Self‐truncated sampling
Negative	Experienced/Described	−0.531 (−0.538)	−0.531 (−0.538)
	Recalled	–	−0.410 [−0.431]
	Judged	−0.535 (−0.670)	−0.437 (−0.565)
Positive	Experienced/Described	0.494 (0.502)	0.494 (0.502)
	Recalled	–	0.404 (0.408)
	Judged	0.567 (0.650)	0.486 (0.550)

*Note*: Each cell shows the mean and median (in parentheses) for a particular combination of direction of experienced/described covariation, type of covariation, and information learning mode.

Since the above analysis did not support an impact of expected covariation on covariation judgment but suggested that the influence of information learning mode on covariation judgment depended on experienced/described covariation, we ran another regression analysis on covariation judgment which discarded expected covariation but added sample size as a predictor to examine whether sample size moderated the observed interaction between information learning mode and experienced/described covariation. Note that all possible two‐way and three‐way interactions were considered in this analysis. Now the best model turned out to be the full model shown below, and it was credibly better than any other nested models (*BF*s >5.26):

JC∼ILM+EDC+SS+ILM×EDC+ILM×SS+EDC×SS+ILM×EDC×SS.

**FIGURE 4:**
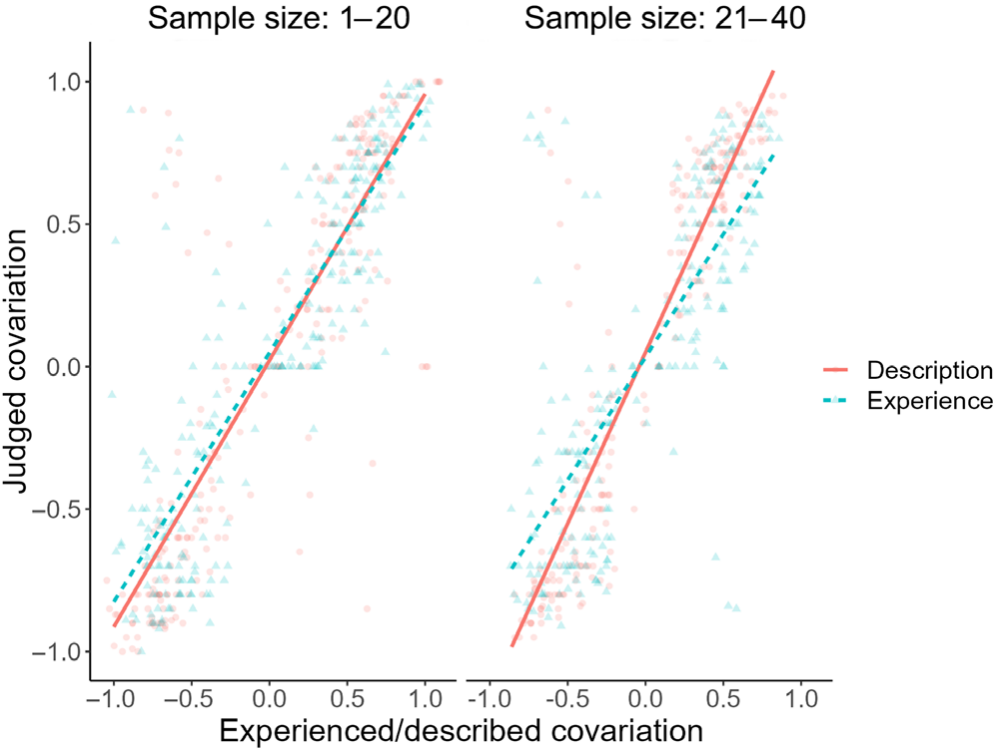
Magnitudes of the description–experience gap in covariation judgment under small (i.e., at most 20 instances) and large (i.e., at least 21 instances) sample sizes in Experiment 1.

According to this model, there was credible three‐way interaction between experienced/described covariation, information learning mode, and sample size (*BF*_incl_ = 30.47, 95% CI = [−0.023, −0.007]). This interaction suggested that the more instances a participant experienced in a trial under the self‐truncated sampling condition, the larger the corresponding description–experience gap would be. See Figure 4 for a graphic illustration of the impact of sample size on the gap.

#### Role of regressive frequency estimation

**FIGURE 5:**
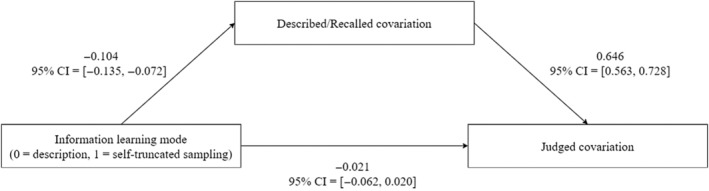
The mediation effect of described/recalled covariation on the description–experience gap in covariation judgment in Experiment 1.

Analyses on experienced and estimated frequencies showed that participants sampled about 20 instances on average under the self‐truncated sampling condition (*M* = 21.54, *Mdn* = 20, 10th percentile = 10, 90th percentile = 37) and demonstrated the typical pattern of regressive estimation. Specifically, such a pattern entailed that cell proportions calculated with estimated frequencies (e.g., 0.3/0.2/0.2/0.3) should be more similar to each other than those calculated with experienced frequencies (e.g., 0.4/0.1/0.1/0.4). To show that this indeed occurred in the current data, we first calculated (as a measure of similarity) the standard deviation of cell proportions for each problem and participant using estimated or experienced frequencies and then compared the results. It turned out that standard deviations regarding estimated frequencies did tend to be smaller than those regarding experienced frequencies (Wilcoxon signed‐rank test, *BF*_10_ = 1.06 × 10^7^, 95% CI of effect size = [−0.487, −0.304]). This regressive frequency estimation naturally led to more moderate recalled covariations relative to experienced covariations (Wilcoxon signed‐rank test, *BF*_10_ = 5.79 × 10^6^, 95% CI of effect size = [−0.561, −0.381]). There was also decisively strong evidence that judged covariation under self‐truncated sampling was associated with recalled covariation (Kendall's tau = 0.627, *BF*_10_ = 7.27 × 10^90^). Finally, results of the mediation analysis between information leaning mode and covariation judgment with described/recalled covariation as the mediator suggested that the description–experience gap was fully mediated by regressive frequency estimates. Specifically, the indirect effect was credibly negative (*M* = −0.067, 95% CI = [−0.090, −0.046]), whereas there was no evidence in support of the direct effect (see Figure 5). Consequently, the total effect was credibly negative (*M* = −0.088, 95% CI = [−0.134, −0.043]), with a proportion mediated of 76.65% (95% CI = [28.33%, 124.98%]).

#### Role of differential weighting of rare instances

The observed gap in covariation judgment might also be interpreted from the perspective of differential weighting of rare instances between different learning modes. On the one hand, judged covariation under description tended to be more extreme than the described one (Wilcoxon signed‐rank test, *Mdn*_diff_ = 0.108, *BF*_10_ = 1.91 × 10^5^, 95% CI of effect size = [0.246, 0.431]), suggesting underweighting of rare instances with regard to their described frequencies. On the other hand, judged covariations under self‐truncated sampling appeared to equal experienced ones (Wilcoxon signed‐rank test, *Mdn*_diff_ = −0.013, *BF*_10_ = 0.325, 95% CI of effect size = [−0.177, 0.002]), suggesting linear weighting of rare instances with regard to their experienced frequencies. Overall, rare instances shown in contingency tables appeared to be more underweighted than those under self‐truncated sampling, in line with the finding of more moderate judgments under self‐truncated sampling. Finally, judged covariation under self‐truncated sampling tended to be more extreme than recalled covariation (Wilcoxon signed‐rank test, *Mdn*_diff_ = 0.235, *BF*_10_ = 3.86 × 10^9^, 95% CI of effect size = [0.424, 0.611]), suggesting underweighting of rare events with regard to estimated frequencies.^4^

### Discussion

Using a range of objective covariations and an other‐yoked design equating described and experienced cell frequencies under respective learning modes, this experiment revealed a description–experience gap in covariation judgment for contingent variables. Specifically, there was a credible interaction between learning mode and described/experienced covariation so that learning by self‐truncated sampling produced more moderate judgment than learning from description given either negative or positive described/experienced covariation. Furthermore, the more extreme the described/experienced covariation was, the larger the gap in covariation judgment would be. These findings were consistent with an account of covariation judgment assuming regressive frequency estimation under self‐truncated sampling. Such regressive estimation was the consequence of memory uncertainty resulting from memory demand and cognitive load under the experience condition. According to this account, cell frequencies estimated from recalled memory under self‐truncated sampling would regress relative to experienced ones. In addition, the more extreme the experienced frequencies were, the stronger the regression effect would be (Fiedler & Unkelbach, 2014).

To the contrary, the influence of regressive diagnosticity under description, if any, appeared to be dominated by the influence of regressive frequency estimation under self‐truncated sampling. Specifically, the full mediation effect of described/recalled covariation in the impact of information learning mode on covariation judgment suggested that the gap was solely produced by regressive frequency estimation. However, the credible three‐way interaction between experienced/described covariation, information learning mode, and sample size in the regression analysis on covariation judgment implied a potential but minor role of regressive diagnosticity in such judgments.

When the same data were analyzed from the perspective of nonlinear weighting of rare instances, it was found that they tended to be underweighted under description but linearly weighted under self‐truncated sampling in terms of their described/experienced frequencies. This result suggested more overweighting of rare events under experience than under description, opposite to the typical pattern found in the research on the description–experience gap in risky choice. This might be the consequence of the similarity between the current covariation judgment task and a risky choice between two risky options. Specifically, each value of one dichotomous variable could co‐occur with either value of the other dichotomous variable in the current task, making it more like a risky choice between two risky options rather than that between a pair of risky and safe options. Note that some (but not all) previous studies on risky choice between two risky options also revealed the pattern of more overweighting of rare events under experience (e.g., Glöckner et al., 2016). When covariation judgment under self‐truncated sampling was analyzed with regard to relevant estimated frequencies, rare instances appeared to be underweighted. This partially counteracted the impact of regressive frequency estimation on covariation judgment. Overall, regressive frequency estimation still appeared to be the major contributing factor for the description–experience gap in covariation judgment.

Note that the results of this study regarding the description–experience gap in covariation judgment could not be accommodated by simply assuming that the description condition constituted an easier environment for covariation judgment and thus led to better performance than the self‐truncated sampling condition. First, participants were not instructed to make such judgments according to any specific quantitative rule. Therefore, participants' responses reflected their subjective assessment of covariation level rather than their attempts to approximate any normative values. Second, even if it was easier for participants under the description condition to make such judgments, it was still unclear why judgments under self‐truncated sampling tended to be more moderate rather than more extreme than those under description. For example, imperfect memory generated by the more difficult self‐truncated sampling condition might trigger participants to rely on recent samples that were less likely to contain rare instances. Consequently, rare instances would appear to be underweighted, leading to more extreme covariation judgment. Finally, using Φ values derived from described/experienced frequencies as the criterion, there was no evidence that covariation judgments under the description condition were more accurate (i.e., closer to the values of the normative measure) than those produced by self‐truncated sampling (Wilcoxon signed‐rank tests, *BF*_10_ = 0.90).

The apparent linear weighting of rare instances under self‐truncated sampling mentioned above suggested that the impact of overestimating the frequencies of rare instances was fully counteracted by the opposite impact of underweighting estimated frequencies. An interesting question to ask was whether the underweighting of estimated frequencies of rare instances might be further enhanced in a covariation‐related decision task. If so, overestimation of the frequencies of rare instances (i.e., regressive frequency estimation) might co‐occur with underweighting of rare instances with regard to their experienced frequencies as in risky choice under experience. If this turned out to be case, then we might not observe a gap in covariation‐related decision, or even find a reversed gap in that self‐truncated sampling led to more extreme responses.

## EXPERIMENT 2

This study was aimed at examining whether a description–experience gap like that observed in covariation judgment also existed in related decisions, or whether an opposite gap might occur due to simultaneous overestimation and underweighting of rare instances. Although decisions are usually built upon relevant judgments, they do not always coincide with each other (e.g., Barron & Yechiam, 2009; Szollosi et al., 2019). Therefore, it is necessary to empirically examine the potential gap in covariation‐related decisions to shed further light on the impacts of information learning mode.

### Method

#### Participants

Seventy‐four students (26 males; *M*_age_ = 21.8 years, range 17–29 years) from Zhejiang University were recruited for this study. Half of the participants took the self‐truncated condition while the other half took the description condition. None of the participants had attended the previous experiment. Participants under the description condition were each paid 6 CNY and those under the self‐truncated sampling condition were each paid 18 CNY. All participants granted their informed consent before taking the study.

#### Materials and procedure

This study was similar to Experiment 1 in terms of experimental materials and design. However, as an experiment aimed at investigating the difference in covariation‐related decision, this study differed in two critical aspects. First, to avoid priming participants to perform the decision task based on their covariation judgment, neither instruction regarding the concept of covariation nor measurement of expected covariation was provided. Otherwise, any difference in a participant's response to the decision task might be a laboratory artifact instead of reflecting a natural tendency in real life. For the same reason, the matching procedure involving description‐based covariation judgment used before was not implemented in this study. Second, the major task in this study was an allocation decision measured by a set of three questions. If participants simply made such decisions based on their covariation judgments, we would expect to observe a gap in such decisions similar to that in covariation judgment. However, if simultaneous overestimation and underweighting of rare instances also occurred in such decisions, an opposite gap might show up.

Take the canteen order scenario as an example. The following instruction was provided under the description condition:

Imagine you are a staff member of the canteen and would like to launch a welfare campaign by offering your customers free meat or vegetable buns. Specifically, for customers ordering soy milk, you can offer a proportion of such customers free meat buns and the remaining customers free vegetable buns. Similarly, for customers ordering milk, you can offer a proportion of such customers free meat buns, and the remaining customers free vegetable buns.

Now, as a staff member of the canteen, you need to decide, compared with customers ordering soy milk, whether you would like to: (1) offer free meat buns instead of vegetable buns to a higher proportion of customers ordering milk; (2) offer free vegetable buns instead of meat buns to a higher proportion of customers ordering milk; or (3) offer free meat or vegetable buns to the same proportion of customers ordering milk. Before you make the decision, you can first collect relevant information by learning about recent records of customers' orders of beverage and food in terms of the number of customers who order each specific combination of beverage and food. When you feel ready for the decision, please report your decision.

The corresponding instruction under the self‐truncated sampling condition was almost the same except that the last sentences were changed into:

Before you make the decision, you can first collect relevant information by learning about recent records of customers' orders of beverage and food. Now you can read the records one‐by‐one, with each record showing the beverage and food ordered by a single customer. When you feel ready for the decision, please stop reading records and report your decision.

After learning about the records (i.e., covariation information) by either description or self‐truncated sampling, participants were required to answer the first two questions shown in the above instruction based on the information they just learned. If a participant responded positively (i.e., responding *yes*) to one of the questions but negatively (i.e., responding *no*) to the other, he or she would be further asked to decide how much the proportion of customers ordering soy milk should differ from the proportion of customers ordering milk in terms of the allocation decision. For example, if the answer to the first question concerning free meat buns was positive, the participant would be asked, compared with customers ordering soy milk, how much higher a proportion of customers ordering milk should be offered free meat buns instead of vegetable buns. Overall, answers to these questions would be combined into a single index of relative contingent preference (RCP) between −1 and 1. By *contingent preference* we mean that the level of preference for offering free meat buns instead of free vegetable buns depends on the type of food a customer ordered, and by *RCP* we mean how much a participant decided the two allocation proportions should differ.

Specifically, if a participant responded positively to the first question, negatively to the second, and stated that the difference in proportion should be 100%, then a value of 1 would be assigned to the index of RCP. This meant that the participant decided to always offer free meat buns to customers ordering milk and always offer free vegetable buns to customers ordering soy milk. If a participant responded negatively to the first question, positively to the second, and stated that the difference in proportion should be 100%, then a value of −1 would be assigned to the index of RCP. This meant that the participant decided to always offer free vegetable buns to customers ordering milk and always offer free meat buns to customers ordering soy milk. For other situations in which responses to the third question were less extreme (i.e., lower than 100%), the value of the index would be set to equal the corresponding response in absolute value. If answers to the first two questions were both negative, the index would be assigned a zero value. This meant that the participant would offer the same proportions of customers ordering soy milk and customers ordering milk free meat or vegetable buns. In summary, the pattern of responses to the first two questions determined the sign of the index, and the answer to the third question determined the magnitude of the index. Finally, after answering the aforementioned questions, participants under the self‐truncated sampling condition were required as before to recall the instances they had experienced and enter their frequency estimates into an empty 2×2 contingency table.

It is worth noting that similar decision tasks had been used in Seggie and Endersby (1972) and Seggie (1987) to investigate whether people had a proper concept of correlation and the potential influential factors. However, their studies involved only a binary decision (i.e., whether to transport a local patient to a distant hospital or not) rather than a quantitative measure of RCP as in the current study. Since the binary decision showed only the direction of RCP but not its magnitude, the quantitative measure adopted in the current study was more informative for revealing the description–experience gap in covariation‐related decision.

#### Data analysis

This study involved the same sequence of analyses as Experiment 1 except for the following two changes. First, the critical dependent variable in this study was RCP (rather than covariation judgment), which measured how much a participant's preference between the two values of a variable would depend on the value of the other variable. Second, because expected covariation was not measured in this study to avoid priming participants to make decisions based on their covariation judgment, no analysis regarding expected covariation was performed.

### Results

#### Description–experience gap

**FIGURE 6:**
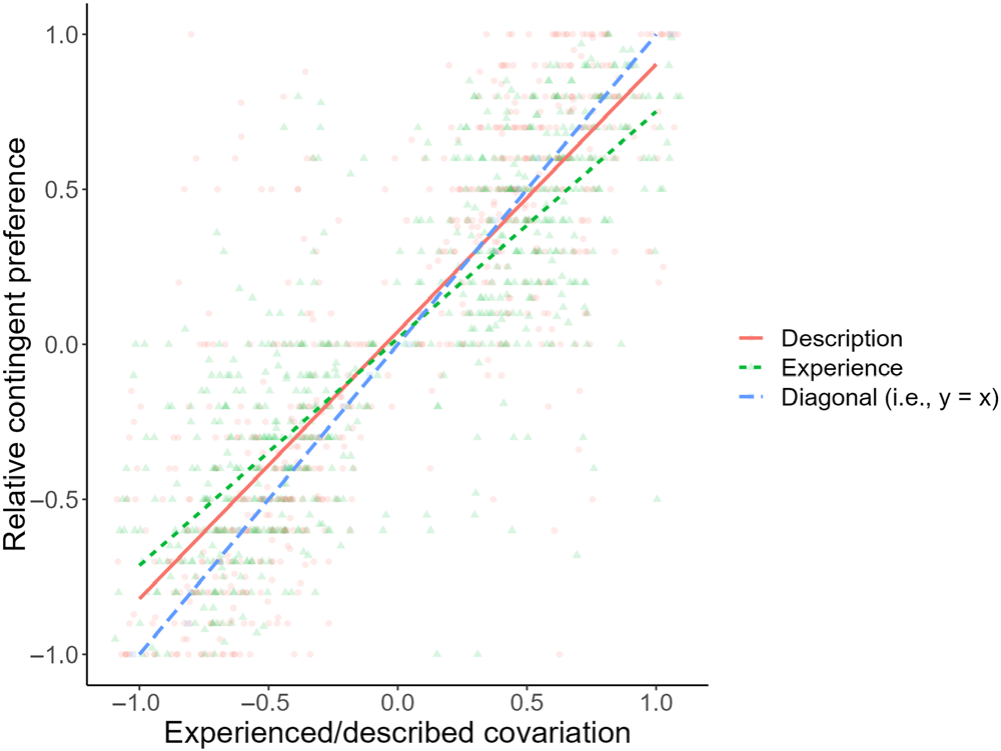
Relative contingent preference under description (red elements) and experience (i.e., self‐truncated sampling; green elements) as functions of described/experienced covariation in Experiment 2.

The regression analysis on RCP showed again that learning by self‐truncated sampling led to more moderate responses than learning from described yoked information. Specifically, the best model contained the main effects of information learning mode and experienced/described covariation as well as their interaction (i.e., RCP ~ ILM + EDC + ILM × EDC). It was credibly better than all the other models considered in the analysis (*BF*s > 13.70). The main effect of information learning mode was not credible (*BF*_incl_ = 0.07, 95% CI of the best model = [−0.057, 0.012]), whereas the interaction was credible (*BF*_incl_ = 95.96, 95% CI of the best model = [−0.190, −0.071]). A regression analysis similar to that in Experiment 1 regarding the impact of sample size on the gap suggested that sample size did not influence the gap in this study. Specifically, the full model (i.e., RCP ~ ILM + EDC + SS + ILM × EDC + ILM × SS + EDC × SS + ILM × EDC × SS) was inferior to the best model without the term of three‐way interaction (*BF*_10_ = 0.007). Figure 6 showed levels of RCP under both learning modes as a function of experienced/described covariation. Clearly the same pattern of difference between the description and self‐truncated sampling conditions was revealed in this study. The more moderate RCP under self‐truncated sampling suggested that rare instances shown in contingency tables were also more underweighted than those under self‐truncated sampling in this decision task. See Table 4 for descriptive statistics of experienced/described and recalled covariations as well as RCP under different information learning modes and directions of experienced/described covariation.

**TABLE 4:** Descriptive statistics of experienced/described and recalled covariation as well as relative contingent preference under different information learning modes and directions of experienced/described covariation in Experiment 2.

Direction of experienced/described covariation	Measure	Information learning mode	
		Description	Self‐truncated sampling
Negative	Experienced/described covariation	−0.543 (−0.528)	−0.543 (−0.528)
	Recalled covariation	–	−0.415 (−0.417)
	Relative contingent preference	−0.450 (−0.500)	−0.385 (−0.400)
Positive	Experienced/described covariation	0.527 (0.500)	0.527 (0.500)
	Recalled covariation	–	0.411 (0.426)
	Relative contingent preference	0.516 (0.500)	0.410 (0.480)

*Note*: Each cell shows the mean and median (in parentheses) for a particular combination of direction of experienced/described covariation, measure, and information learning mode.

#### Role of regressive frequency estimation

**FIGURE 7:**
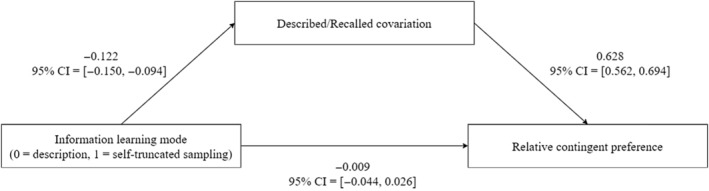
The mediation effect of described/recalled covariation on the description–experience gap in relative contingent preference in Experiment 2.

Analyses on experienced and estimated frequencies showed that participants again sampled about 20 instances on average under the self‐truncated sampling condition (*M* = 19.80, *Mdn* = 20, 10th percentile = 10, 90th percentile = 31) and demonstrated the typical pattern of regressive estimation. Specifically, standard deviations regarding estimated frequencies tended to be smaller than those regarding experienced frequencies (Wilcoxon signed‐rank test, *BF*_10_ = 1.29 × 10^18^, 95% CI of effect size = [−0.693, −0.533]). Consequently, recalled covariations appeared more moderate than experienced covariations (Wilcoxon signed‐rank test, *BF*_10_ = 3.23 × 10^9^, 95% CI of effect size = [−0.588, −0.430]). Finally, there was very strong evidence that level of RCP under self‐truncated sampling was associated with recalled covariation (Kendall's tau = 0.650, *BF*_10_ = 8.91 × 10^134^). Results of the mediation analysis suggested again that the description–experience gap was fully mediated by regressive frequency estimates. Specifically, the indirect effect was credibly negative (*M* = −0.076, 95% CI = [−0.096, −0.058]), whereas there was no clear evidence in support of the direct effect (see Figure 7). Consequently, the total effect was credibly negative (*M* = −0.085, 95% CI = [−0.123, −0.048]), with a proportion mediated of 89.54% (95% CI = [45.87%, 133.21%]).

### Discussion

This study extended our examination of the impact of information learning mode on covariation judgment to covariation‐related decision. According to Crocker's (1981) normative process model of covariation judgment, such judgment would set the foundation for subsequent decisions. Consequently, the same pattern of more moderate responses under self‐truncated sampling was expected. However, research on the description–experience gap in risky choice suggested that, when making a decision instead of judgment, overestimation of rare instances could co‐occur with underweighting of rare instances, potentially leading to an opposite gap in covariation‐related decision. The pattern of more moderate RCP under self‐truncated sampling revealed in this study supported Crocker's model instead of simultaneous overestimation and underweighting of rare instances. As mentioned earlier, this might be partly due to the similarity between the covariation context involved in our research and a risky choice between two risky options instead of that between a safe option and a risky option. Furthermore, as in Experiment 1, the results of mediation analysis did not support the role of regressive diagnosticity in the related decision. Overall, the current research showed that self‐truncated sampling would lead to more moderate response than the description condition with regard to both covariation judgment and related decision and suggested a major role of regressive frequency estimation in such description–experience gaps.

## GENERAL DISCUSSION

Covariation information can be learned either from a summary description in the form of a contingency table with well‐calibrated cell frequencies or from experiencing individual instances showing values of potentially contingent variables. When learning the information from experience, people usually have some control over the amount of information in that they can decide when to stop the information search. However, this self‐truncated sampling condition for covariation judgment and related decision has been rarely examined and never directly compared with the description condition in the literature. To fill this gap, we conducted a series of experiments involving both the description and self‐truncated sampling conditions. These studies consistently showed that learning covariation information by self‐truncated sampling led to more moderate covariation judgment and related decision than learning the same information from description. This difference constitutes yet another form of description–experience gap in judgment and decision‐making beyond those in risky choice and other tasks (e.g., Fantino & Navarro, 2012). Furthermore, memory error under self‐truncated sampling in terms of regressive frequency estimation appeared to play a critical role in shaping the gaps. The regressive frequency estimation was likely to be the consequence of the enhanced memory uncertainty resulting from memory demand and cognitive load under the specific information learning mode. It was also found that rare instances under description were consistently more underweighted than those under self‐truncated sampling, regardless of the nature of the task involved. This pattern was at odds with the typical one found in research on the description–experience gap in risky choice but echoed the finding in some recent studies on risky choice between two risky options.

Echoing Crocker's (1981) normative process of covariation judgment, the current research suggested a critical role of regressive frequency estimation from recalled memory in covariation judgment and related decision. Our studies, however, did not explicitly manipulate recalled memory and then observe the resultant covariation judgment and related decision. In other words, the implication about the role of recalled memory was derived from correlational rather than causal evidence. Consequently, there are alternative explanations of the major findings from our studies. For example, it might be the case that regressive frequency estimation and more moderate covariation judgment under self‐truncated sampling were produced by distinct cognitive underpinnings, and recalled memory was not the proximate cause of covariation judgment. Specifically, covariation judgment under self‐truncated sampling could be the product of a belief‐updating process (e.g., Hogarth & Einhorn, 1992) independent of recalled memory, whereas memory error revealed in the recall task was produced by the enhanced memory uncertainty under the self‐truncated sampling condition. Finally, covariation‐related decision might be built upon covariation judgment, leading to the same pattern of description–experience gap in related decision in terms of RCP. Although it is currently unclear how a belief‐updating process (or similarly an associative learning process) under self‐truncated sampling could lead to the observed gaps, future research should try to distinguish between these alternative accounts. Memory processes contributing to the regressive pattern of frequency estimation shall also be examined.

Another issue to be addressed is the role of differential weighting of cell information in the gaps. Differential patterns of probability weighting have been invoked to explain the description–experience gap in risky choice, and unequal weighting of cell information has also been frequently used to explain deficits in covariation judgment. The regressive frequency estimation revealed in the current research suggested an overestimation of rare instances, and the resultant frequency estimation might then be integrated for covariation judgment. In Experiment 1, which measured covariation judgment, judged covariations tended to be more extreme than the recalled ones given correlated pairs of dichotomous variables. This difference suggested an underweighting of estimated frequencies of rare instances in making covariation judgment, which could counteract the potential impact of regressive frequency estimation on the relevant gap. Similar differences between described and judged covariations under the description condition were also found in the current research. In other words, underweighting of described and recalled rare instances appeared to exist under respective conditions and exert similar influences on covariation judgment. Consequently, underweighting of relevant information appeared to contribute little to the observed gaps, and the regressive frequency estimation under self‐truncated sampling remained a major candidate for the cognitive underpinning of the relevant gaps. Nevertheless, for a deeper understanding of underlying cognitive mechanisms, future research can examine factors influencing the relative impact of overestimating rare instances against underweighting their estimated frequencies under the self‐truncated sampling condition. Factors determining how much rare instances under description are underweighted in covariation judgment shall also be examined.

Optional stopping, that is, stopping information search based on properties observed in the sampled data (e.g., Wulff et al., 2018; Wulff & Pachur, 2016) might also play a role in experience‐based covariation judgment and related decision and thus the relevant gaps. Studies on impression formation under self‐truncated sampling (e.g., Prager et al., 2018) suggested that participants under this condition might stop information search when the available information offers a clear‐cut picture of the target person. A similar mechanism might exist for covariation judgment in the sense that people would stop sampling when the available information suggests a level of covariation consistent with their expectation. If expected covariations tended to be more moderate than experienced covariations, this stopping rule might produce the observed gaps. This account requires further that expected covariation is related to judged covariation under self‐truncated sampling. For Experiment 1, which measured expected covariation, relevant analyses supported the precondition that expected covariations were more moderate than experience covariations. For example, absolute value of expected covariation under the self‐truncated sampling condition in Experiment 1 tended to be smaller than that of experienced covariation (expected: *M* = 0.380, *Mdn* = 0.350; experienced: *M* = 0.546, *Mdn* = 0.577, *BF*_10_ = 1.68 × 10^7^, 95% CI of effect size = [−0.402, −0.272]). However, the requirement that expected covariation should be related to judged covariation was not supported by the current data according to the major regression analyses on the gaps. Therefore, this rule of optional stopping does not seem to contribute to the observed gaps. Future research can further investigate other optional stopping rules that might contribute to the gaps.

## CONFLICT OF INTEREST STATEMENT

The authors both declare no potential conflicts of interest about the current research.

## ETHICS STATEMENT

All experiments were ethically approved by the ethics committee of the Department of Psychology and Behavioral Sciences, Zhejiang University, and all participants signed informed consent forms before experiments.

## Data Availability

Data and materials for the experiments reported in this manuscript can be accessed via https://osf.io/k64cp/.

## Endnotes

^1^
Covariation, contingency, and correlation are three distinct terms that are often used in the literature to indicate the same idea of an association between two events or variables. When necessary, we follow the cited articles for the choice of term in the current article.

^2^
A similar procedure (i.e., free sampling) was also adopted in research on experience‐based risky choice and pseudocontingency. Both free sampling and self‐truncated sampling allowed participants to determine the size of the experienced sample, but only the former permitted participants to decide which option or context to sample at each trial (e.g., Bott & Meiser, 2020; Hertwig et al., 2004).

^3^
For a small proportion (about 4%) of formal trials under the self‐truncated sampling condition, participants sampled the population of instances exhaustively and thus might not have stopped the information search at their will. However, excluding such problems from the reported analyses led to virtually the same results in this and the next experiments.

^4^
A similar study using a wider range of objective covariations and a self‐yoked (i.e., within‐subject) design regarding information learning mode led to virtually the same results.

## References

[pchj703-bib-0001] Alloy, L. B. , & Tabachnik, N. (1984). Assessment of covariation by humans and animals: The joint influence of prior expectations and current situational information. Psychological Review, 91, 112–149. 10.1037/0033-295X.91.1.112 6571422

[pchj703-bib-0002] Arkes, H. R. , & Harkness, A. R. (1983). Estimates of contingency between two dichotomous variables. Journal of Experimental Psychology: General, 112(1), 117–135. 10.1037/0096-3445.112.1.117

[pchj703-bib-0004] Barron, G. , & Yechiam, E. (2009). The coexistence of overestimation and underweighting of rare events and the contingent recency effect. Judgment and Decision Making, 4(6), 447–460. 10.1017/S1930297500003983

[pchj703-bib-0005] Beyth‐Marom, R. (1982). Perception of correlation reexamined. Memory & Cognition, 10(6), 511–519. 10.3758/BF03202433

[pchj703-bib-0006] Bott, F. M. , & Meiser, T. (2020). Pseudocontingency inference and choice: The role of information sampling. Journal of Experimental Psychology: Learning, Memory, and Cognition, 46(9), 1624–1644. 10.1037/xlm0000840 32297791

[pchj703-bib-0007] Bürkner, P. (2017). Brms: An R package for Bayesian multilevel models using Stan. Journal of Statistical Software, 80(1), 1–28. 10.18637/jss.v080.i01

[pchj703-bib-0008] Camilleri, A. R. , & Newell, B. R. (2011). When and why rare events are underweighted: A direct comparison of the sampling, partial feedback, full feedback and description choice paradigms. Psychonomic Bulletin & Review, 18(2), 377–384. 10.3758/s13423-010-0040-2 21327342

[pchj703-bib-0009] Chapman, L. J. , & Chapman, J. P. (1969). Illusory correlation as an obstacle to the use of valid psychodiagnostic signs. Journal of Abnormal Psychology, 74(3), 271–280. 10.1037/h0027592 4896551

[pchj703-bib-0010] Crocker, J. (1981). Judgment of covariation by social perceivers. Psychological Bulletin, 90(2), 272–292. 10.1037/0033-2909.90.2.272

[pchj703-bib-0011] Fantino, E. , & Navarro, A. (2012). Description–experience gaps: Assessments in other choice paradigms. Journal of Behavioral Decision Making, 25(3), 303–314. 10.1002/bdm.737

[pchj703-bib-0012] Fiedler, K. (2010). Pseudocontingencies can override genuine contingencies between multiple cues. Psychonomic Bulletin & Review, 17(4), 504–509. 10.3758/PBR.17.4.504 20702869

[pchj703-bib-0013] Fiedler, K. , & Freytag, P. (2004). Pseudocontingencies. Journal of Personality and Social Psychology, 87, 453–467. 10.1037/0022-3514.87.4.453 15491271

[pchj703-bib-0014] Fiedler, K. , Freytag, P. , & Meiser, T. (2009). Pseudocontingencies: An integrative account of an intriguing cognitive illusion. Psychological Review, 116(1), 187–206. 10.1037/a0014480 19159153

[pchj703-bib-0015] Fiedler, K. , & Kareev, Y. (2006). Does decision quality (always) increase with the size of information samples? Some vicissitudes in applying the law of large numbers. Journal of Experimental Psychology: Learning, Memory, and Cognition, 32(4), 883–903. 10.1037/0278-7393.32.4.883 16822155

[pchj703-bib-0016] Fiedler, K. , Renn, S.‐Y. , & Kareev, Y. (2010). Mood and judgments based on sequential sampling. Journal of Behavioral Decision Making, 23(5), 483–495. 10.1002/bdm.669

[pchj703-bib-0017] Fiedler, K. , & Unkelbach, C. (2014). Regressive judgment: Implications of a universal property of the empirical world. Current Directions in Psychological Science, 23(5), 361–367. 10.1177/0963721414546330

[pchj703-bib-0018] Fleig, H. , Meiser, T. , Ettlin, F. , & Rummel, J. (2017). Statistical numeracy as a moderator of (pseudo)contingency effects on decision behavior. Acta Psychologica, 174, 68–79. 10.1016/j.actpsy.2017.01.002 28189707

[pchj703-bib-0019] Glöckner, A. , Hilbig, B. E. , Henninger, F. , & Fiedler, S. (2016). The reversed description‐experience gap: Disentangling sources of presentation format effects in risky choice. Journal of Experimental Psychology: General, 145(4), 486–508. 10.1037/a0040103 26974209

[pchj703-bib-0020] Hamilton, D. L. , Dugan, P. M. , & Trolier, T. K. (1985). The formation of stereotypic beliefs: Further evidence for distinctiveness‐based illusory correlations. Journal of Personality and Social Psychology, 48(1), 5–17. 10.1037/0022-3514.48.1.5

[pchj703-bib-0021] Hamilton, D. L. , & Gifford, R. (1976). Illusory correlations in interpersonal perception: A cognitive basis of stereotypic judgments. Journal of Experimental Social Psychology, 12, 392–407. 10.1016/S0022-1031(76)80006-6

[pchj703-bib-0022] Hamilton, D. L. , & Rose, T. L. (1980). Illusory correlation and the maintenance of stereotypic beliefs. Journal of Personality and Social Psychology, 39(5), 832–845. 10.1037/0022-3514.39.5.832

[pchj703-bib-0023] Hertwig, R. , Barron, G. , Weber, E. U. , & Erev, I. (2004). Decisions from experience and the effect of rare events in risky choice. Psychological Science, 15(8), 534–539. 10.1111/j.0956-7976.2004.00715.x 15270998

[pchj703-bib-0025] Hogarth, R. M. , & Einhorn, H. J. (1992). Order effects in belief updating: The belief‐adjustment model. Cognitive Psychology, 24(1), 1–55. 10.1016/0010-0285(92)90002-J

[pchj703-bib-0026] JASP Team . (2023). JASP (Version 0.17.1)[Computer software].

[pchj703-bib-0027] Jenkins, H. M. , & Ward, W. C. (1965). Judgment of contingency between responses and outcomes. Psychological Monographs: General and Applied, 79(1), 1–17. 10.1037/h0093874 14300511

[pchj703-bib-0028] Kao, S.‐F. , & Wasserman, E. A. (1993). Assessment of an information integration account of contingency judgment with examination of subjective cell importance and method of information presentation. Journal of Experimental Psychology: Learning, Memory, and Cognition, 19(6), 1363–1386. 10.1037/0278-7393.19.6.1363

[pchj703-bib-0032] Kruschke, J. K. , & Liddell, T. M. (2018). Bayesian data analysis for newcomers. Psychonomic Bulletin & Review, 25(1), 155–177. 10.3758/s13423-017-1272-1 28405907

[pchj703-bib-0033] Lindley, D. V. (1965). Introduction to probability and statistics from a Bayesian point of view, part 2: Inference. Cambridge University Press.

[pchj703-bib-0034] Mata, A. (2016). Judgment of covariation: A review. Psicologia, 30(1), 61–74. 10.17575/rpsicol.v30i1.1082

[pchj703-bib-0035] Mercier, P. , & Parr, W. (1996). Inter‐trial interval, stimulus duration and number of trials in contingency judgments. British Journal of Psychology, 87(4), 549–566. 10.1111/j.2044-8295.1996.tb02608.x

[pchj703-bib-0036] Nisbett, R. E. , & Ross, L. (1980). Human inference: Strategies and shortcomings of social judgment. Prentice‐Hall.

[pchj703-bib-0037] Prager, J. , & Fiedler, K. (2021). Forming impressions from self‐truncated sampling of traits – Interplay of Thurstonian and Brunswikian sampling effects. Journal of Personality and Social Psychology, 121, 474–497. 10.1037/pspa0000274 34807699

[pchj703-bib-0038] Prager, J. , Krueger, J. I. , & Fiedler, K. (2018). Towards a deeper understanding of impression formation‐new insights gained from a cognitive‐ecological perspective. Journal of Personality and Social Psychology, 115(3), 379–397. 10.1037/pspa0000123 29975075

[pchj703-bib-0039] R Core Team . (2022). R: A language and environment for statistical computing. R Foundation for Statistical Computing URL https://www.Rproject.org/

[pchj703-bib-0040] Rouder, J. N. , Speckman, P. L. , Sun, D. , Morey, R. D. , & Iverson, G. (2009). Bayesian t tests for accepting and rejecting the null hypothesis. Psychonomic Bulletin & Review, 16(2), 225–237. 10.3758/PBR.16.2.225 19293088

[pchj703-bib-0041] Seggie, I. (1987). The judgment of covariation between binary variables: Some conditions that influence the process. Memory & Cognition, 15(4), 341–348. 10.3758/BF03197036 3670054

[pchj703-bib-0042] Seggie, J. L. , & Endersby, H. (1972). The empirical implications of piaget's concept of correlation. Australian Journal of Psychology, 24(1), 3–8. 10.1080/00049537208255778

[pchj703-bib-0044] Shaklee, H. (1983). Human covariation judgment: Accuracy and strategy. Learning and Motivation, 14, 433–448. 10.1016/0023-9690(83)90026-7

[pchj703-bib-0045] Shaklee, H. , & Mims, M. (1982). Sources of error in judging event covariations: Effects of memory demands. Journal of Experimental Psychology: Learning, Memory, and Cognition, 8(3), 208–224. 10.1037/0278-7393.8.3.208

[pchj703-bib-0046] Shaklee, H. , & Tucker, D. (1980). A rule analysis of judgments of covariation between events. Memory & Cognition, 8(5), 459–467. 10.3758/BF03211142 7442548

[pchj703-bib-0047] Smedslund, J. (1963). The concept of correlation in adults. Scandinavian Journal of Psychology, 4(1), 165–173. 10.1111/j.1467-9450.1963.tb01324.x

[pchj703-bib-0048] Szollosi, A. , Liang, G. , Konstantinidis, E. , Donkin, C. , & Newell, B. R. (2019). Simultaneous underweighting and overestimation of rare events: Unpacking a paradox. Journal of Experimental Psychology: General, 148(12), 2207–2217. 10.1037/xge0000603 31033320

[pchj703-bib-0049] Tingley, D. , Yamamoto, T. , Hirose, K. , Keele, L. , & Imai, K. (2014). Mediation: R package for causal mediation analysis. Journal of Statistical Software, 59, 1–38. 10.18637/jss.v059.i05 26917999

[pchj703-bib-0051] Vogel, T. , Freytag, P. , Kutzner, F. , & Fiedler, K. (2013). Pseudocontingencies derived from categorically organized memory representations. Memory & Cognition, 41, 1185–1199. 10.3758/s13421-013-0331-8 23740145

[pchj703-bib-0052] Ward, W. C. , & Jenkins, H. M. (1965). The display of information and the judgment of contingency. Canadian Journal of Psychology, 19(3), 231–241. 10.1037/h0082908 5824962

[pchj703-bib-0053] Wasserman, E. A. , Dorner, W. W. , & Kao, S.‐F. (1990). Contributions of specific cell information to judgments of interevent contingency. Journal of Experimental Psychology: Learning, Memory, and Cognition, 16, 509–521. 10.1037/0278-7393.16.3.509 2140406

[pchj703-bib-0054] Wulff, D. U. , Mergenthaler‐Canseco, M. , & Hertwig, R. (2018). A meta‐analytic review of two modes of learning and the description‐experience gap. Psychological Bulletin, 144(2), 140–176. 10.1037/bul0000115 29239630

[pchj703-bib-0055] Wulff, D. U. , & Pachur, T. (2016). Modeling valuations from experience: A comment on Ashby and Rakow (2014). Journal of Experimental Psychology: Learning, Memory, and Cognition, 42(1), 158–166. 10.1037/xlm0000165 26751013

[pchj703-bib-0056] Yates, J. F. , & Curley, S. P. (1986). Contingency judgment: Primacy effects and attention decrement. Acta Psychologica, 62(3), 293–302. 10.1016/0001-6918(86)90092-2 3766201

[pchj703-bib-0057] Yechiam, E. , & Busemeyer, J. R. (2006). The effect of foregone payoffs on underweighting small probability events. Journal of Behavioral Decision Making, 19, 1–16. 10.1002/bdm.509

